# Spatiotemporal Dynamics of Dengue Epidemics, Southern Vietnam

**DOI:** 10.3201/eid1906.121323

**Published:** 2013-06

**Authors:** Hoang Quoc Cuong, Nguyen Thanh Vu, Bernard Cazelles, Maciej F. Boni, Khoa T.D. Thai, Maia A. Rabaa, Luong Chan Quang, Cameron P. Simmons, Tran Ngoc Huu, Katherine L. Anders

**Affiliations:** Oxford University Clinical Research Unit, Ho Chi Minh City, Vietnam (H.Q. Cuong, M.F. Boni, K.T.D. Thai, C.P. Simmons, K.L. Anders);; Pasteur Institute, Ho Chi Minh City (H.Q. Cuong, N.T. Vu, L.C. Quang, T.N. Huu);; University of Oxford, Oxford, UK (M.F. Boni, C.P. Simmons, K.L. Anders);; Unités Mixtes de Recherche, Paris, France (B. Cazelles);; L'Unité Mixte Internationale, Bondy, France (B. Cazelles);; University Medical Centre, Rotterdam, the Netherlands (K.T.D. Thai);; Pennsylvania State University, University Park, Pennsylvania, USA (M.A. Rabaa);; Monash University, Melbourne, Victoria, Australia (K.L. Anders)

**Keywords:** Dengue, epidemiology, surveillance, transmission, heterogeneity, spatial distribution, Vietnam, arthropod vectors, viruses, vector-borne infections

## Abstract

An improved understanding of heterogeneities in dengue virus transmission might provide insights into biological and ecologic drivers and facilitate predictions of the magnitude, timing, and location of future dengue epidemics. To investigate dengue dynamics in urban Ho Chi Minh City and neighboring rural provinces in Vietnam, we analyzed a 10-year monthly time series of dengue surveillance data from southern Vietnam. The per capita incidence of dengue was lower in Ho Chi Minh City than in most rural provinces; annual epidemics occurred 1–3 months later in Ho Chi Minh City than elsewhere. The timing and the magnitude of annual epidemics were significantly more correlated in nearby districts than in remote districts, suggesting that local biological and ecologic drivers operate at a scale of 50–100 km. Dengue incidence during the dry season accounted for 63% of variability in epidemic magnitude. These findings can aid the targeting of vector-control interventions and the planning for dengue vaccine implementation.

Dengue is a growing international public health problem for which a licensed vaccine, therapeutic drugs, and effective vector control programs are lacking. The increasing number of cases is associated with an expanding geographic range and increasing intensity of transmission in affected areas (*1*,*2*). The dynamics of dengue in disease-endemic areas are characterized by strong seasonality and multiannual epidemic peaks (*3*), with substantial interannual and spatial heterogeneity in the magnitude of seasonal epidemics (*4*). Extrinsic factors, including climatic and environmental variables, have been hypothesized to drive annual seasonality; intrinsic factors associated with human host demographics, population immunity, and the virus, drive the multiannual dynamics (*5*–*7*). Analyses from Southeast Asia have demonstrated multiannual oscillations in dengue incidence (*8*–*10*), which have been variably associated with macroclimatic weather cycles (exemplified by the El Niño Southern Oscillation) in different settings and with changes in population demographics in Thailand (*11*). In Thailand, a spatiotemporal analysis showed that the multiannual cycle emanated from Bangkok out to more distant provinces (*9*).

Knowledge of spatial and temporal patterns in dengue incidence at a subnational level is relevant for 2 main reasons: it can provide insights into the biological and ecologic mechanisms that drive transmission, and it might facilitate predictions of the magnitude, timing, and location of future dengue epidemics. For both of these reasons, detailed spatial resolution is useful because aggregated datasets can obscure some of the factors that influence the timing and size of individual local epidemics.

In southern Vietnam, dengue occurs year-round; a marked seasonal peak occurs during the rainy months of June–December, and the number of cases has been increasing over the past 15 years (*12*). As in many dengue-endemic settings, the dengue surveillance system in Vietnam relies on passive reporting of clinically diagnosed dengue in hospitalized patients. Vector control is the primary tool available for dengue prevention and control. In Vietnam, vector control is pursued through a targeted approach of low-volume space spraying of households around clusters of reported dengue cases. This strategy faces limitations in timeliness and sensitivity because of the reliance on and response to case reports for hospitalized patients only. A predictive epidemiologic tool that enables prioritization of limited resources for the most cost-effective reduction in cases would be highly valued in dengue-endemic settings.

To investigate spatial and temporal trends for dengue in southern Vietnam, we used a monthly time series of dengue surveillance data over 10 years, disaggregated to the district level. We analyzed the periodicity of dengue incidence, determined whether annual epidemics consistently originate in and spread from Ho Chi Minh City (HCMC) or another location, and characterized the differences in the magnitude and timing of epidemics among provinces and districts.

## Methods

### Study Area and Data Sources

**Figure 1:**
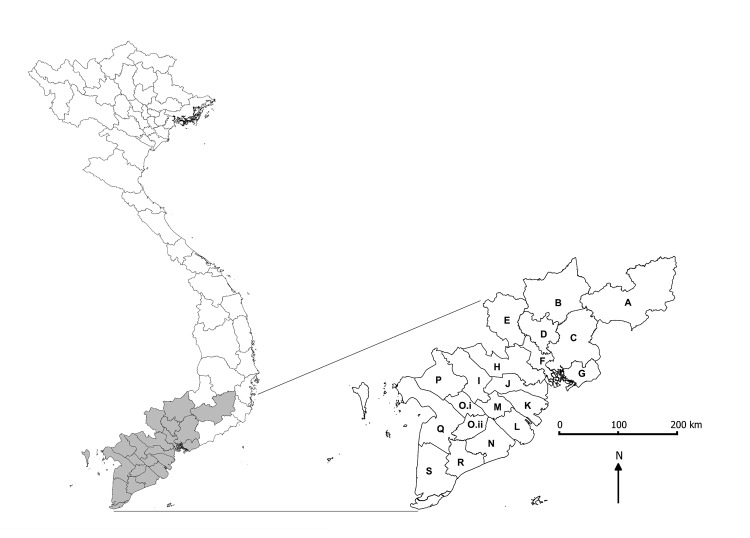
Vietnam and the southern 19 provinces included in this analysis. The map shows current administrative boundaries; for our analysis, we aggregated 2 provinces (Can Tho and Hau Giang) to reflect the administrative boundaries before 2004. A, Lam Dong; B, Binh Phuoc; C, Dong Nai; D, Binh Duong; E, Tay Ninh; F, Ho Chi Minh City; G, Ba Ria – Vung Tau; H, Long An; I, Dong Thap; J, Tien Giang; K, Ben Tre; L, Tra Vinh; M, Vinh Long; N, Soc Trang; O, Can Tho; P, An Giang; Q, Kien Giang; R, Bac Lieu; S, Ca Mau.

Administrative boundaries for the southern region of Vietnam in 2001 were used for consistency across the study period (2001–2010): this region included 19 provinces, subdivided into 159 districts (Figure 1). In 2009, the total population of the study area was 32.3 million (≈38% of the national population). Demographic data were obtained from the Government Statistics Office (*13*).

As part of the national dengue control program in Vietnam, dengue case notifications in southern Vietnam are aggregated by provincial authorities and reported monthly to the Pasteur Institute, Ho Chi Minh City (PI-HCMC). Only hospitalized dengue patients are reported, and the case definition is a clinical diagnosis of dengue at hospital discharge. A conservative estimate of the specificity of a clinical dengue case diagnosis in Vietnam is ≈50%, based on IgM in 1 serum sample collected from a small proportion (<10%) of patients (PI-HCMC, unpub. data). Most cases are not laboratory confirmed.

The time series used in this analysis included all dengue cases reported from January 1, 2001, through December 31, 2010, from the 19 provinces of southern Vietnam; cases were aggregated by month of hospital admission and district of residence. No identifying personal information was included in the data. The study was approved by the institutional review board of PI-HCMC.

### Determining Dengue Periodicity

To explore the periodicity in the dengue incidence time series, we performed continuous wavelet transform, which decomposes the time series into time and frequency components. Calculation of the wavelet power spectrum quantifies the distribution of the variance of the time series in the time–frequency domain (*14*,*15*). The Morlet wavelet was used, and all analyses were performed with MATLAB software version 6.5 (MathWorks Inc., Natick, MA, USA). All time series were square-root transformed and normalized, and the trend was suppressed before analysis by removing periodic components >6 years with a classical low-pass filter (*16*). Significance levels were computed with an appropriate bootstrapping scheme that used the Markov process and preserved the short-term temporal correlation of the raw series, the HMM Surrogate (*17*); 1,000 HMM Surrogate series were used. Significance was set at p<0.05.

### Quantifying Synchrony 

To explore the temporal relationship between dengue time series across the 19 provinces and 159 districts, we subjected each time series to wavelet decomposition as described above. Using the imaginary and the real parts of the wavelet transform in the annual mode (0.8*–*1.2 years), we computed the phase angles and phase difference between 2 time series (*18*). The wavelet decomposition was also used as a band-pass filter for filtering the raw time series in the annual mode to obtain the seasonal oscillations, which were used together with the phase differences for computing the pairwise delay (in days) between district and province dengue time series (*8*, equation 8).

We used Spearman and Pearson correlation tests to assess whether larger dengue epidemics were more synchronous, as defined by the variance in pairwise interprovince or interdistrict delays as described above. Additionally, pairwise correlations between district-level and province-level dengue time series were made in 3 transformed datasets: square root–transformed monthly incidence normalized to a mean of 0 and SD of 1 (a correlation in magnitude and timing of dengue epidemics), square root–transformed and normalized annual incidence (a correlation in magnitude only), and phase angles (a correlation in timing only). The relationship between these correlation coefficients and the intervening distance between provinces or districts was assessed by using the nonparametric spline covariance function from the NCF (spatial nonparametric covariance function) package in R (*19*) (R 2.14.2, R Foundation for Statistical Computing, Vienna, Austria) with 1,000 bootstraps to generate 95% confidence bands. We calculated pairwise distances between districts and provinces by using geographic coordinates of district and province centroids in R. To test the hypothesis that similarity in the timing and/or magnitude of dengue epidemics increases with spatial proximity, we performed a Mantel test of the correlation between each matrix of coefficients above and the intervening distance between provinces and districts (in km), by using the NCF package in R (*19*).

### Predicting Seasonal Epidemic Magnitude

To determine whether the magnitude of a seasonal dengue epidemic could be predicted by the dengue activity in the previous interepidemic period, we used a linear model of log-transformed dengue incidence during the epidemic period (April–December) in each district or province as a function of the incidence during the preceding dry period (January–March) in the same district or province. These definitions of epidemic and dry periods were decided a priori and were based on scrutiny of the seasonal pattern of dengue across the study period; in HCMC, these periods were shifted (a priori) 1 month later (May–January and February–April, respectively) to account for the fact that the trough in dengue incidence occurred markedly later in HCMC than in other provinces.

## Results

### Temporal Trends 

**Figure 2:**
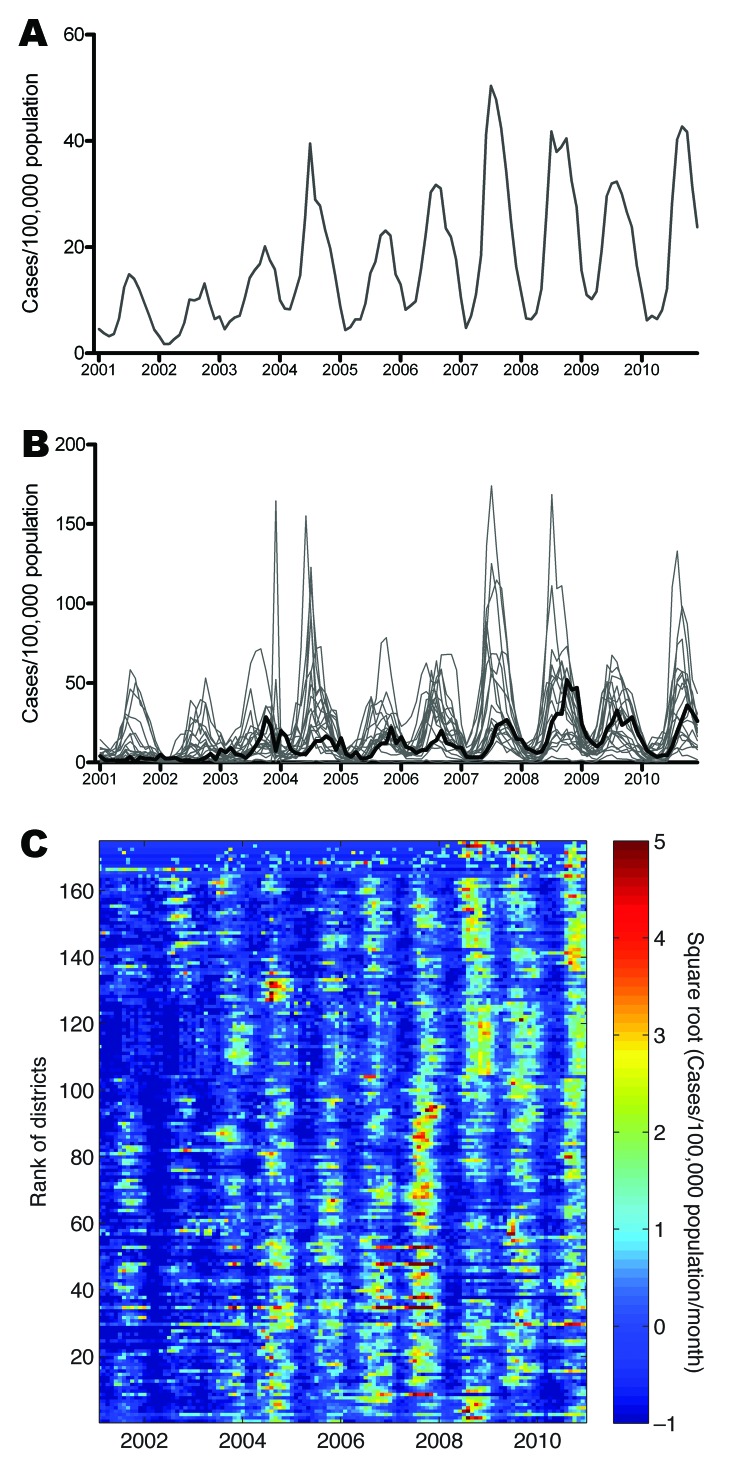
Dengue time series from the 19 provinces and 159 districts in southern Vietnam, 2001–2010. A) Monthly aggregate time series of dengue cases reported from provinces. B) Monthly dengue incidence in each province; **boldface** line indicates Ho Chi Minh City. C) Monthly dengue incidence in each district. Data have been square-root transformed and normalized to zero mean and unit variance. Districts are ordered from north (top) to south (bottom) by first ordering provinces north to south, then ordering districts within each province, according to latitude of district centroid.

During 2001–2010, a total of 592,938 dengue cases were reported from the southern 19 provinces of Vietnam; median was 66,608 cases annually (range 22,519–88,311 cases). This finding corresponds to the median annual incidence of 232 cases per 100,000 population (annual range 78–288 cases/100,000 population). Most cases (mean 82%, annual range 74%–92%) were reported during the rainy season, June–December (Figure 2, panel A). Differences in temporal trends between the provinces are apparent in Figure 2, panel B, which shows that in terms of per capita incidence, substantially higher epidemic peaks are reached in provinces outside HCMC than within HCMC. The annual peak also appears consistently later in HCMC than in other provinces. The time series for individual provinces are shown in the online Technical Appendix Figure 1 (wwwnc.cdc.gov/EID/article/19/12-1323-Techapp1.pdf).

 A visual comparison of time series between the 159 districts (Figure 2, panel C) suggests overall seasonal synchrony across southern Vietnam but with geographic differences in the timing and magnitude of high-incidence periods at the district level. We explored whether the high dengue incidence outside HCMC represented urban transmission in provincial cities and towns, but we found no parametric or nonparametric correlation between district-level cumulative 10-year dengue incidence and either the proportion of the district population that was rural/urban (p>0.6) or the district population density (p>0.1).

### Dengue Periodicity 

**Figure 3:**
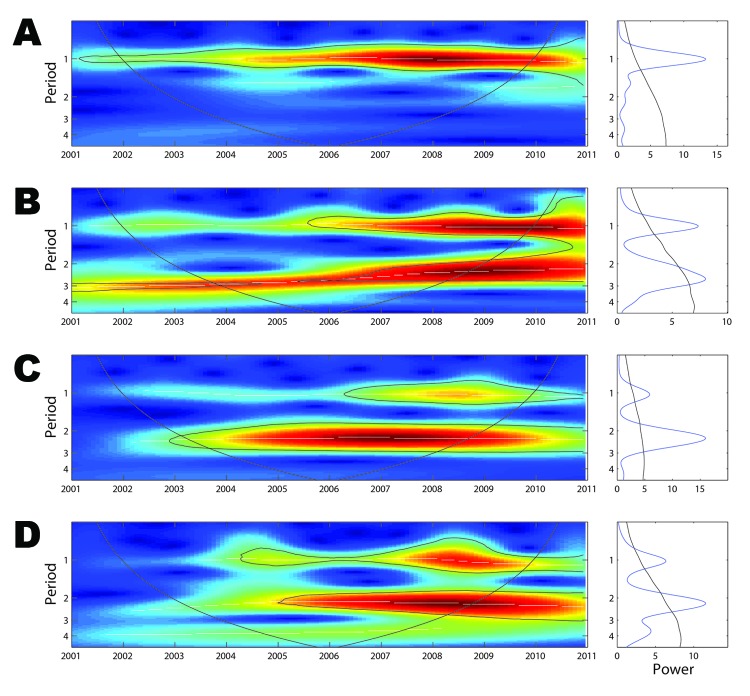
Wavelet analysis of dengue periodicity, 2001–2010. A) Left panel: wavelet power spectrum (WPS) of the aggregate monthly dengue time series for southern Vietnam (square-root transformed, normalized, and trend suppressed). Colors code for increasing spectrum intensity, from blue to red; dotted lines show statistically significant area (threshold of 95% CI); the black curve delimits the cone of influence (region not influenced by edge effects). Right panel: Mean spectrum (solid line) with its threshold value of 95% CI (dotted line) for the aggregate time series. B) WPS and mean spectrum for Binh Duong Province. C) WPS and mean spectrum for Bac Lieu Province. D) WPS and mean spectrum for Ca Mau Province. The wavelet power spectra for Binh Duong, Bac Lieu, and Ca Mau Provinces are shown because they were the only 3 provinces in which a dominant multiannual cycle was detected.

Wavelet analysis of the aggregate time series showed a strong annual periodicity but no multiannual cycle (Figure 3, panel A). To investigate spatial differences in dengue periodicity, we performed wavelet analyses for individual province time series (online Technical Appendix Figure 2). An annual cycle was apparent in all provinces but with substantial heterogeneity in the relative strength of the multiannual component. In 3 provinces, a 2–3 year multiannual cycle was either dominant (Bac Lieu and Ca Mau, Figure 3, panels C and D), or of similar intensity as the annual signal (Binh Duong, Figure 3, panel B). In several other provinces, a transient subdominant multiannual cycle was observed, but these cycles are difficult to interpret epidemiologically.

### Origins and Spread of Annual Dengue Epidemics

**Figure 4:**
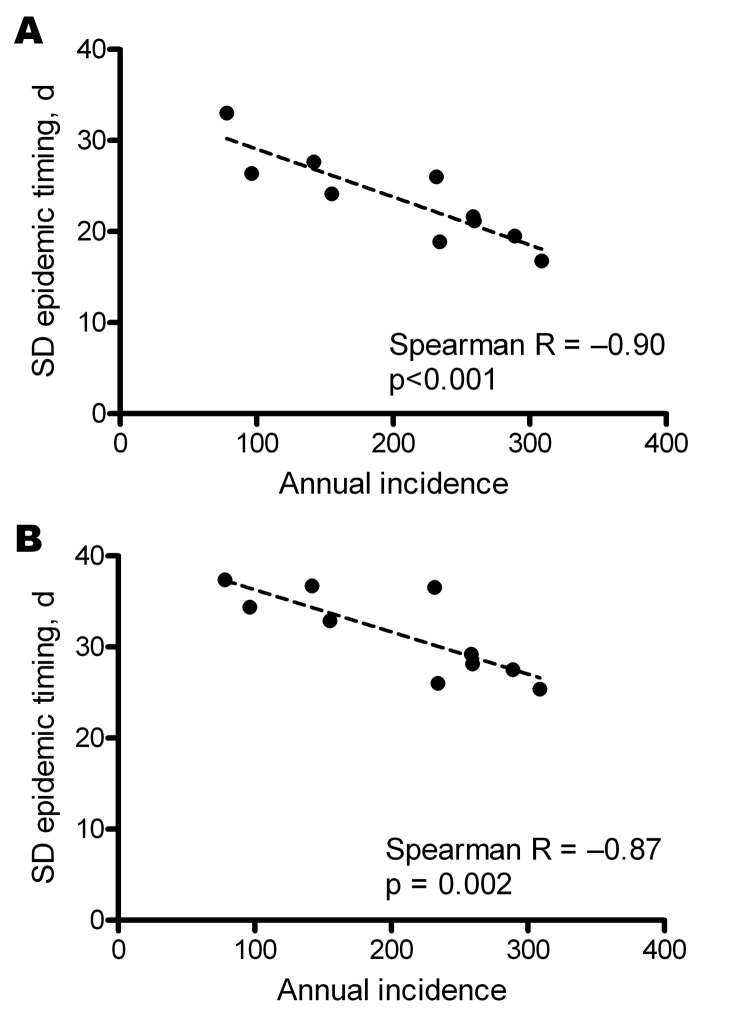
Correlation across provinces (A) or districts (B) between annual dengue incidence and variation in epidemic timing. Epidemic timing represents the pairwise interprovince or interdistrict delay between wavelet transformed annual dengue time series. The variation in epidemic timing is significantly correlated with the overall magnitude of transmission in that year; there is less variation (i.e., more synchrony) in the timing of dengue epidemics across southern Vietnam in high-incidence years than in low-incidence years

Despite the pronounced seasonality of dengue, we observed substantial heterogeneity in the timing of annual epidemics across the study region and period. The average interval between the province experiencing the earliest and latest dengue epidemic within a given year was 14.2 weeks (annual range 8.9–20.0 weeks). The interprovince and interdistrict lag in the onset of seasonal dengue epidemics was significantly negatively correlated with the overall magnitude of the epidemic (Figure 4); in other words, dengue epidemics are significantly more synchronous throughout the region in years with higher overall incidence than in years with lower incidence.

**Figure 5:**
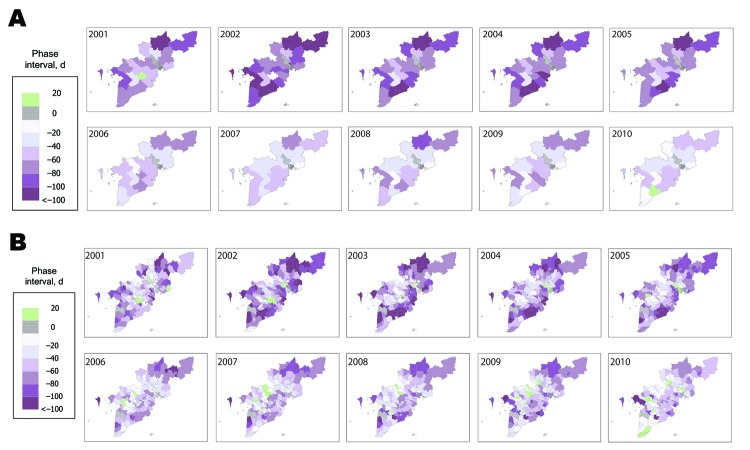
Spatiotemporal patterns in annual dengue epidemics in southern Vietnam. The phase interval (days) between the dengue time series in each province (A) relative to Ho Chi Minh City (HCMC) and each district (B) relative to District 1 in HCMC is shown by year. The largest negative values (dark purple) indicate the earliest locations for the annual dengue epidemics, zero (gray hatched) represents synchrony with the HCMC time series, and positive values (green) indicate dengue epidemics that occurred later than in HCMC.

To explore further the observation that annual dengue epidemics occur later in HCMC than elsewhere, we plotted the phase interval (in days) between the dengue time series in each province relative to HCMC, averaged across the 10-year period (not shown) and in each individual year (Figure 5, panel A). A multifocal origin of seasonal dengue epidemics in southern Vietnam was revealed, in which the epidemic cycle in each of the 18 provinces preceded HCMC by a median of 55 days (range 26–90 days) averaged over the 10-year period. The dengue epidemic occurred later in HCMC than in all other provinces in all but 2 years (2001 and 2010), and in these 2 years, in only 1 province did the dengue epidemic occur later than in HCMC. Figure 5, panel B, shows the equivalent analysis for district-level time series. These analyses indicated that in some locations (Binh Phuoc to the north of HCMC, Lam Dong to the northwest, Soc Trang in the far south, and Kien Giang in the southwest), despite their considerable distance from one another, dengue epidemics were consistently among the earliest each year (Figure 1). However, the earliest epidemics often occur in multiple simultaneous locations, and there is no clear spatial pattern for the movement of the dengue epidemic within a given season. Signals of early epidemics in Lam Dong should be treated with caution because the case numbers for the dry and the rainy seasons were small.

### Synchrony in Dengue Dynamics

**Figure 6:**
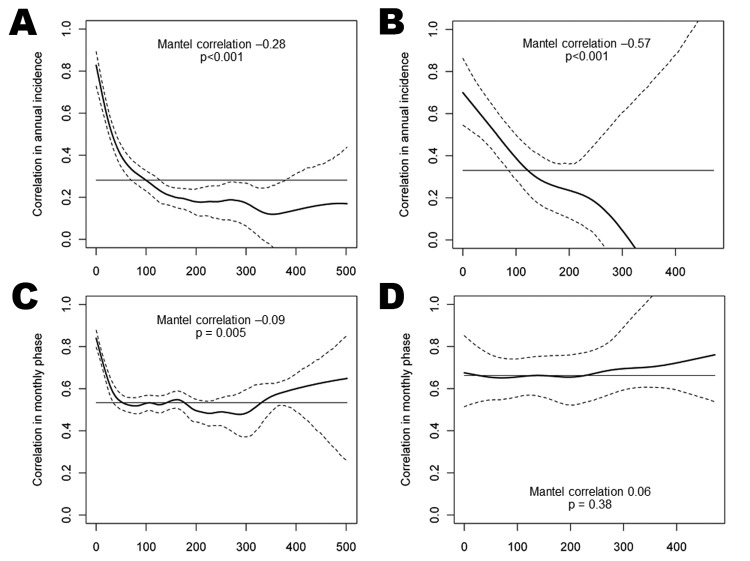
Spatial coherence in the magnitude (A and B) and timing (C and D) of dengue epidemics in southern Vietnam. District data are shown in panels A and C, and province data in panels B and D. Solid lines represent the correlation between provinces/districts as a function of the distance between the centroids of those provinces/districts, in kilometers. Dashed lines represent 95% CIs, and the horizontal line is the overall correlation across southern Vietnam. Coherence in the magnitude and timing of epidemics was measured by pairwise correlation between provinces/districts in their standardized square root–transformed annual incidence and monthly phase series, respectively.

Overall, dengue epidemics across southern Vietnam were more highly correlated in timing than in incidence (Figure 6, horizontal lines), consistent with the pronounced seasonality of dengue virus (DENV) transmission despite heterogeneities in epidemic magnitude. Coherence in the size of annual dengue epidemics was spatially dependent (p<0.001 for provinces and districts; Mantel test). Districts within 100 km of each other were significantly more likely to have concurrent high-incidence and low-incidence years (Figure 6, panel A), and the degree of correlation increased with increasing proximity. This spatial dependence was observed also for province-level data (Figure 6, panel B) out to 122 km. The timing of dengue epidemic cycles was less spatially dependent. Nearby districts experienced more synchronous epidemics (p = 0.005), significant out to 52 km (Figure 6, panel C); however, this spatial dependence was not seen at the province level (Figure 6, panel D; p = 0.38). Spatially dependent synchrony out to 101 km was also observed when correlating the raw monthly time series, which takes the timing and the magnitude of dengue epidemics into account (Technical Appendix Figure 3). Overall, this analysis demonstrates spatial clustering of dengue activity at a scale of up to ≈50–100 km.

### Dry Season Dengue as Predictor of Subsequent Epidemic Magnitude

**Figure 7:**
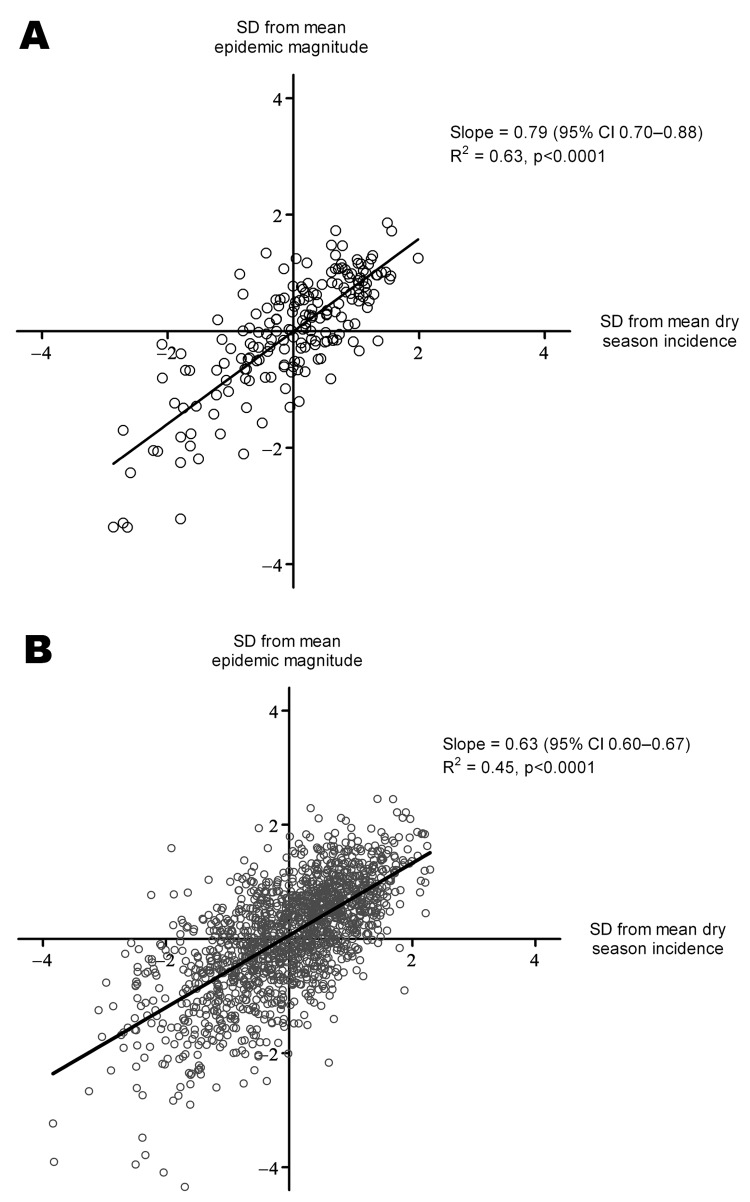
Dry season dengue incidence as a predictor of the magnitude of the subsequent dengue epidemic. Plots show the association between annual epidemic incidence (April–December) and the preceding dry season dengue incidence (January–March). For Ho Chi Minh City (HCMC), these definitions were a priori shifted 1 month later (May–January and February–April, respectively) because of the consistently later occurrence of the dengue epidemic season in HCMC. Each point represents 1 province (A) or district (B) and year, correlating the standard deviation from mean incidence in the rainy season against the standard deviation from mean incidence in the preceding dry season, in the same province or district. The solid line shows fitted values from a linear model of epidemic incidence against dry season incidence. We excluded 71 data points from the district analysis (B) because there were no dengue cases during the dry season.

We found a significant positive association between dengue incidence during the dry season and the magnitude of the subsequent dengue epidemic in a given province or district. Using province-level data, we found that an increase of 1 SD above the mean dengue incidence during the dry season was associated with an increase of 0.79 SDs (95% CI 0.70–0.88; p<0.0001) above the mean epidemic magnitude during the subsequent rainy season (Figure 7, panel A). Dry season incidence accounted for 63% of the variation in epidemic magnitude among provinces and years. Stratified by province, this association held for 12 of the 19 provinces (data not shown); this finding might reflect a lack of power to detect such an association with 10 data points. Stratified by year, the association was significant (p<0.001) for every year during 2001–2010. Using data for 159 districts, we found that the association between dry season and wet season incidence was also highly significant (p<0.001) overall (Figure 7, panel B) and stratified by province or year (not shown), although the proportion of the total variation in epidemic magnitude accounted for by dry season incidence was lower (45%).

## Discussion

In southern Vietnam, dengue exhibits pronounced seasonal peaks that coincide with the rainy season and causes tens of thousands of hospitalizations every year. Within this overall high-transmission setting, substantial spatial and temporal heterogeneity is apparent from our monthly district-level time series analysis. Several characteristics of the epidemic cycle in this setting could help inform public health efforts to prevent and control dengue.

All provinces in southern Vietnam exhibit annual seasonality; however, 2 provinces in the far south (Bac Lieu and Ca Mau) and 1 north of HCMC (Binh Duong) also show evidence of multiannual cycles. These patterns suggest possible differences in the intrinsic and extrinsic drivers of DENV transmission in these provinces; however, interpretation of these findings must take into account the limitations of wavelet analysis, especially within a 10-year time series, in which it is difficult to obtain strong statistical support for long multiannual cycles. Further considerations in the interpretation of our findings relate to the limitations of dengue surveillance data as a proxy for DENV transmission. A variable majority of DENV infections are asymptomatic (*20*), and it is possible that the observed disease dynamics are an imperfect reflection of the underlying DENV transmission dynamics. Furthermore, case surveillance data for dengue, as for many diseases, have sensitivity and specificity limitations, because of underreporting and a lack of laboratory confirmation, respectively.

Dengue is typically thought of as an urban disease (*1*,*21*–*23*). HCMC is the major urban center in southern Vietnam, but the per capita incidence reached there during seasonal dengue peaks is substantially lower than in most of the other less urban provinces. This finding supports evidence from Cambodia (*24*), Thailand (*25*), and Vietnam (*26*) that dengue presents a health challenge in periurban and rural settings as well as in urban centers. Furthermore, dengue epidemics occur ≈2–3 months later in HCMC than in the surrounding rural provinces, indicating that HCMC cannot act as a source population initiating annual epidemics in other provinces. In fact, the presence of multiple locations with early dengue epidemics indicates that there may not be a consistent year-to-year spatial pattern of DENV transmission and no consistent geographic source from which dengue epidemics emanate. These findings lead directly to new research questions for dengue in southern Vietnam, to explore what factors influence the timing and size of the annual epidemic wave in each province.

Although the dynamics we describe give the appearance of dengue traveling from several early foci to HCMC each season, we think this is unlikely for 3 reasons. First, dengue cases occur throughout the dry season in HCMC as well as in other provinces; hence, re-introduction of DENV is not required to initiate the seasonal increase in cases. Second, we found no correlation between the timing of dengue epidemics in each province or district and their geographic distance from HCMC (analysis not shown); such a correlation would have suggested a traveling wave of infection toward HCMC (*9*,*10*). Third, phylogenetic analyses of DENV-1 (*27*) and DENV-2 (*28*) from southern Vietnam suggest that these viruses disperse from HCMC out to other provinces. Our findings are not necessarily inconsistent with these phylogenetic studies; the former relate to spatiotemporal dynamics in case incidence within 1 dengue season, and the latter describe processes of viral dispersion over several years. Together, these studies suggest that despite a lower per capita incidence, the larger absolute virus population in HCMC exerts an influence on the relatively small virus populations in rural provinces but that other conditions determine the timing and magnitude of the increased transmission during annual dengue epidemics.

Several factors could explain the observed spatial dynamics besides the geographic movement of DENV. First, vector development, survival, and biting behavior and viral replication within the vector are all highly sensitive to climatic conditions including rainfall, temperature, and relative humidity (*29*,*30*), and it is possible that geographic differences in microclimate might contribute to differences in the timing of dengue epidemics across southern Vietnam. Second, the ratio of vectors to human hosts, rather than density of vectors or hosts alone, has been shown to be a key parameter in classical models of vector-borne disease transmission (*31*,*32*) and in epidemiologic studies (*26*). Understanding how this ratio differs between HCMC and lower population-density areas might help explain the lower per capita incidence in HCMC. Third, the later and lower-incidence seasonal epidemics in HCMC are consistent with the higher median age of the population in HCMC (*13*); an older, and thus more immune, population reduces the probability of a vector feeding on a susceptible or infectious person, both of which are necessary to drive transmission.

We demonstrate that the timing and the magnitude of annual dengue epidemics in southern Vietnam are significantly more similar in districts in closer proximity, and this association remains significant out to 50–100 km. This finding suggests a role for local drivers of DENV transmission operating at this spatial scale, possibly including microclimatic and environmental determinants of vector abundance and vector–host contact, population immunity, or human movement patterns at the scale of a district or town. Several studies have demonstrated focal transmission of DENV at a fine spatial scale from 100 m to 1 km, attributable to direct chains of transmission, vector flight distances, human movement, and serotype-specific immune profiles (*33*–*35*). We extended this finding by demonstrating spatial dependence of dengue incidence at an intermediate scale of <100 km, with a weakening association as proximity decreases. This association is comparable with the spatial extent of synchrony (180 km) demonstrated among province-level dengue time series in Thailand (*9*). This finding highlights the need to analyze disease surveillance data at as fine a spatial scale as possible because the spatial dependence of dengue epidemic timing was not apparent in our province-level analysis.

In Vietnam, the public health authority classifies dengue incidence within any administrative boundary as epidemic when cases exceed 2 SDs above the mean incidence in that month and location over the past 5 years (excluding any previous epidemic months). This definition allows little to no lead time for intervention, and the ability to predict further in advance where epidemic thresholds are likely to be crossed could improve the timeliness and possibly the effectiveness of control interventions. Our simple result showing that dengue incidence during the dry interepidemic period accounts for 63% of variability in rainy season dengue epidemic magnitude might help local public health authorities take advantage of dengue’s predictable cyclical behavior for informing public health action. This method relies on using data that are available 3–6 months before the peak of DENV transmission, and this extra lead time might be an invaluable resource for targeted dengue intervention planning in years when a season with a large number of dengue cases is expected. Evaluating the performance of this model in real time for forecasting dengue epidemic magnitude is the next challenge in determining its public health utility.

Dengue prevention and control activities in many disease-endemic settings, including Vietnam, currently rely on targeted spraying of adulticides to reduce vector populations in and around the homes of reported patients. These activities are usually complemented with public health outreach and some routine activities to reduce vector breeding sites, within the constraints of limited public health budgets. Future intervention strategies will also incorporate rollout of a dengue vaccine (*36*,*37*) or modified mosquitoes (*38*). Understanding the spatial dynamics and timing of dengue epidemics might enhance the implementation of current and future interventions by improved targeting to avert high-incidence dengue seasons based on dry-season signals and to dampen dengue incidence in neighboring areas.

Technical Appendix Dengue in southern Vietnam, 2001–2010. Time series of incidence, by province. Wavelet analysis of periodicity, by province; and spatial synchrony in magnitude and timing of annual dengue epidemics, at district and province levels.
