# Supplementary material for: Spatiotemporal Dynamics of Dengue Epidemics, Southern Vietnam
Source: Emerg Infect Dis. 2013 Jun;19(6):945–53. doi: 10.3201/eid1906.121323 (PMC3713821; doi:10.3201/eid1906.121323)
Supplement: Technical Appendix — Dengue in southern Vietnam, 2001–2010. Time series of incidence, by province. Wavelet analysis of periodicity, by province; and spatial synchrony in magnitude and timing of annual dengue epidemics, at district and province levels. [file 12-1323-Techapp-s1.pdf]

Article DOI: http://dx.doi.org/10.3201/eid1906.121323

Spatiotemporal Dynamics of Dengue Epidemics, Southern Vietnam

Technical Appendix

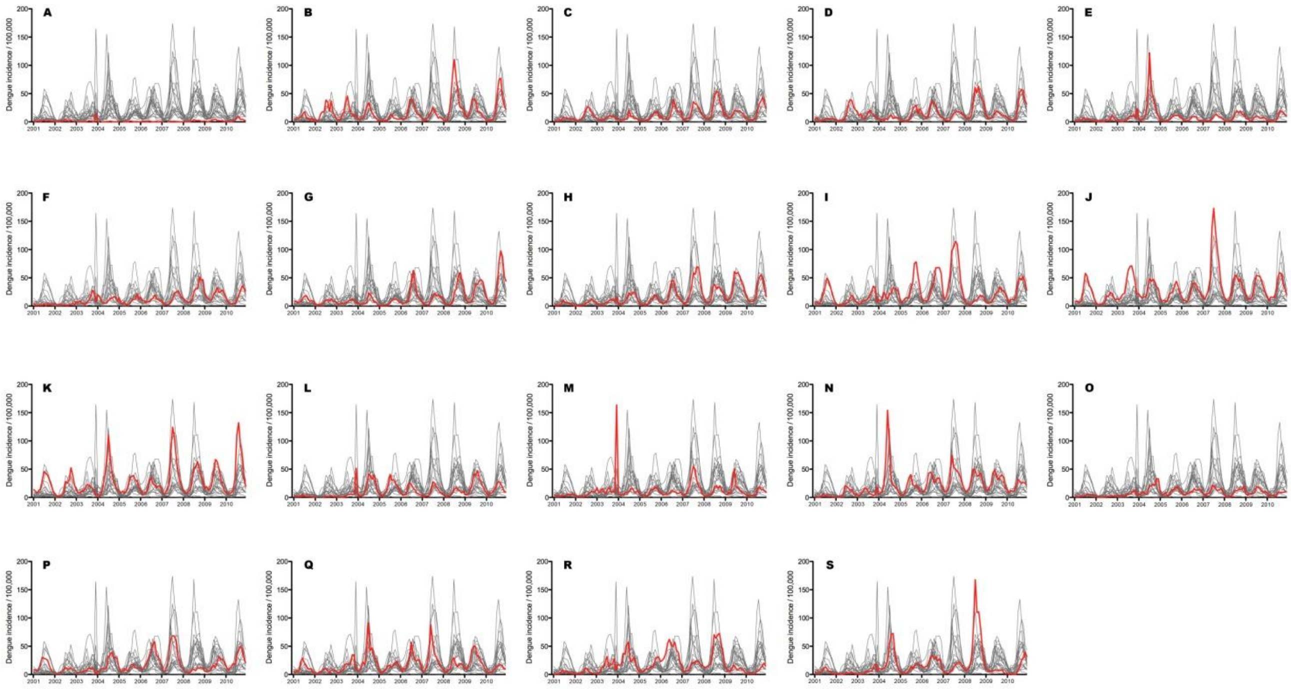

Technical Appendix Figure 1. Time series of dengue incidence in southern Vietnam, 2001–2010, by province. Each panel shows the time series of an individual province highlighted against the other provincial time series. Key to provinces: A, Lam Dong; B, Binh Phuoc; C, Dong Nai; D, Binh Duong; E, Tay Ninh; F, Ho Chi Minh City; G, Ba Ria – Vung Tau; H, Long An; I, Dong Thap; J, Tien Giang; K, Ben Tre; L, Tra Vinh; M, Vinh Long; N, Soc Trang; O, Can Tho; P, An Giang; Q, Kien Giang; R, Bac Lieu; S, Ca Mau.

Technical Appendix Figure 2 (following pages). Wavelet analysis of dengue periodicity, 2001–2010, by province. Left panel: the wavelet power spectrum of monthly dengue cases in provincial time series (square-root transformed, normalized, and trend suppressed); colors code for increasing spectrum intensity, from blue to red; dotted lines show statistically significant area (threshold of 95% CI); the black curve delimits the cone of influence (region not influenced by edge effects). Right panel: mean spectrum (solid line) with its threshold value of 95% CI

(dotted line). Key to provinces: A, Lam Dong, B; Binh Phuoc; C, Dong Nai; D, Binh Duong; E, Tay Ninh; F, Ho Chi Minh City; G, Ba Ria – Vung Tau; H, Long An; I, Dong Thap; J, Tien Giang; K, Ben Tre; L, Tra Vinh; M, Vinh Long; N, Soc Trang; O, Can Tho; P, An Giang; Q, Kien Giang; R, Bac Lieu; S, Ca Mau.

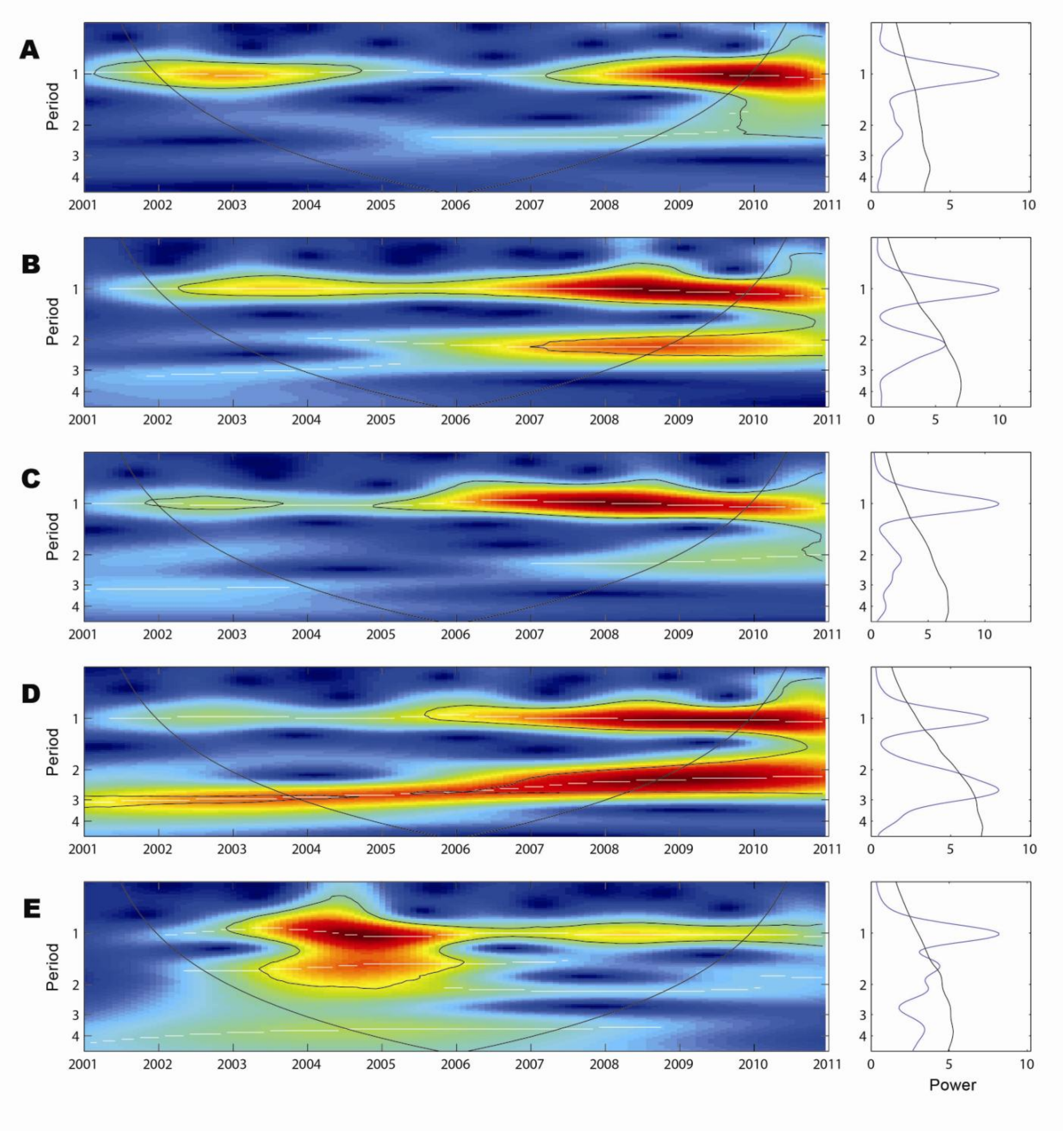

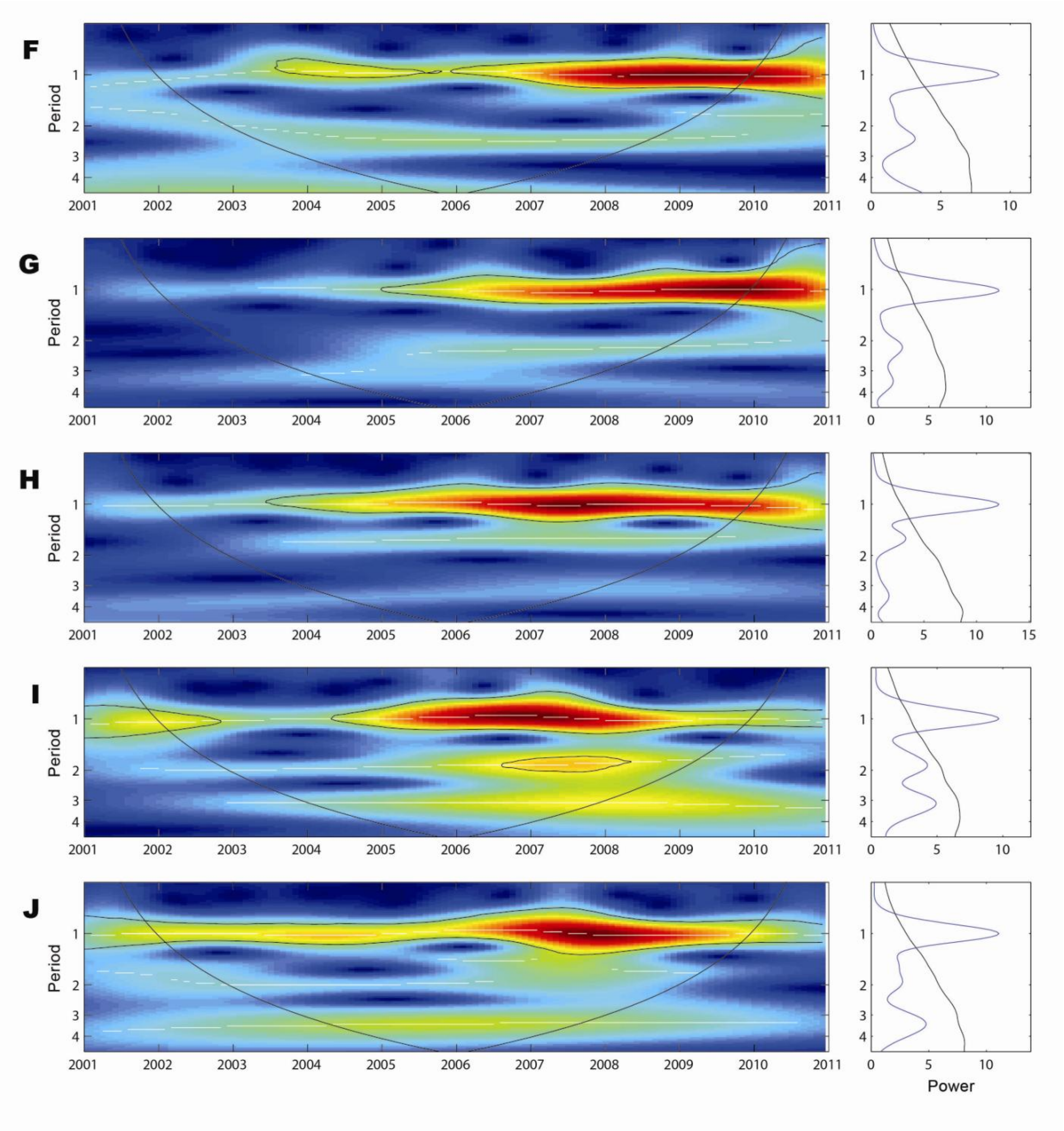
F
G
H
I
J
Period
1
2
3
4
2001
2002
2003
2004
2005
2006
2007
2008
2009
2010
2011
0
5
10
15
Power

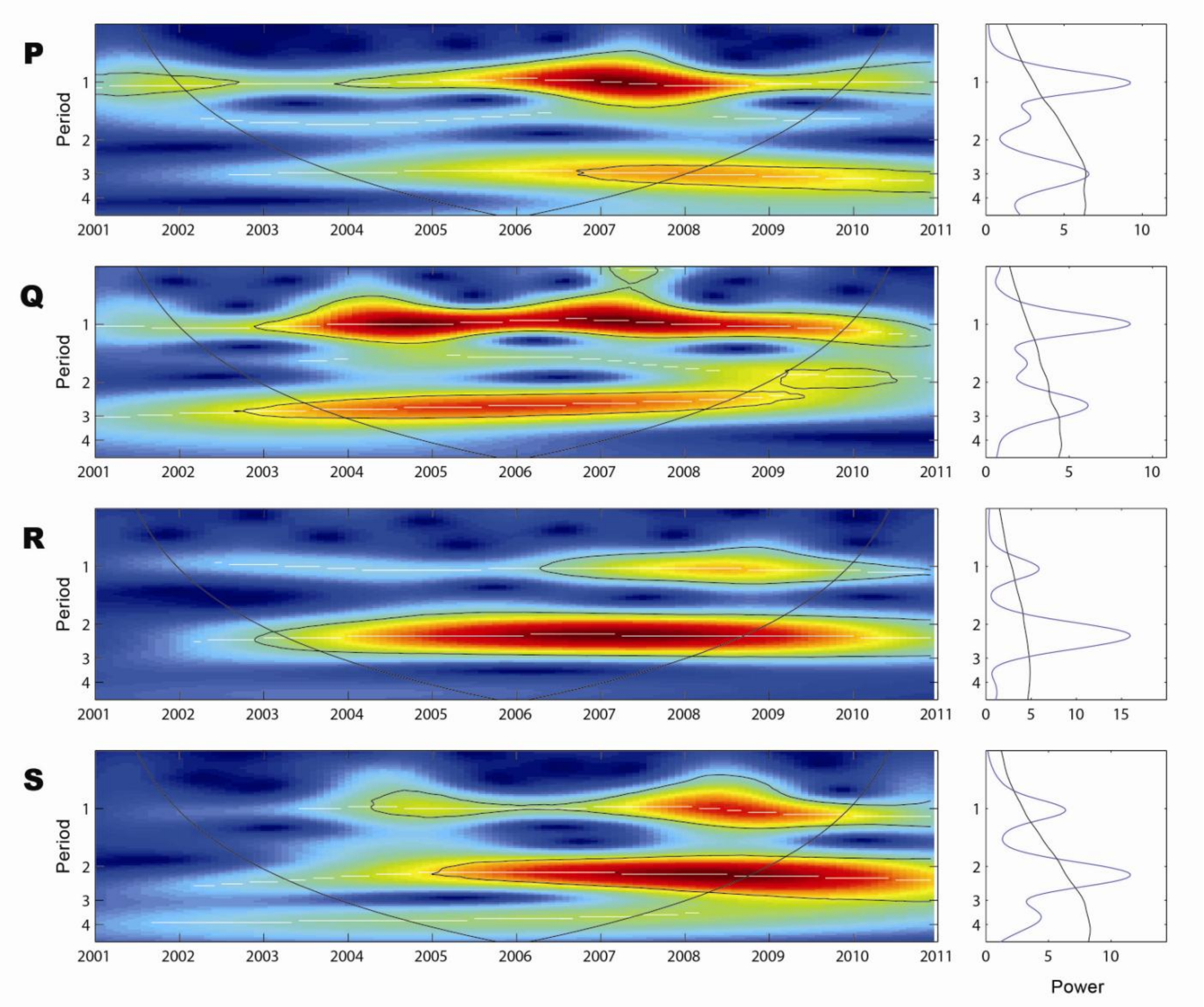
P
Q
R
S
Period
Power
2001
2002
2003
2004
2005
2006
2007
2008
2009
2010
2011

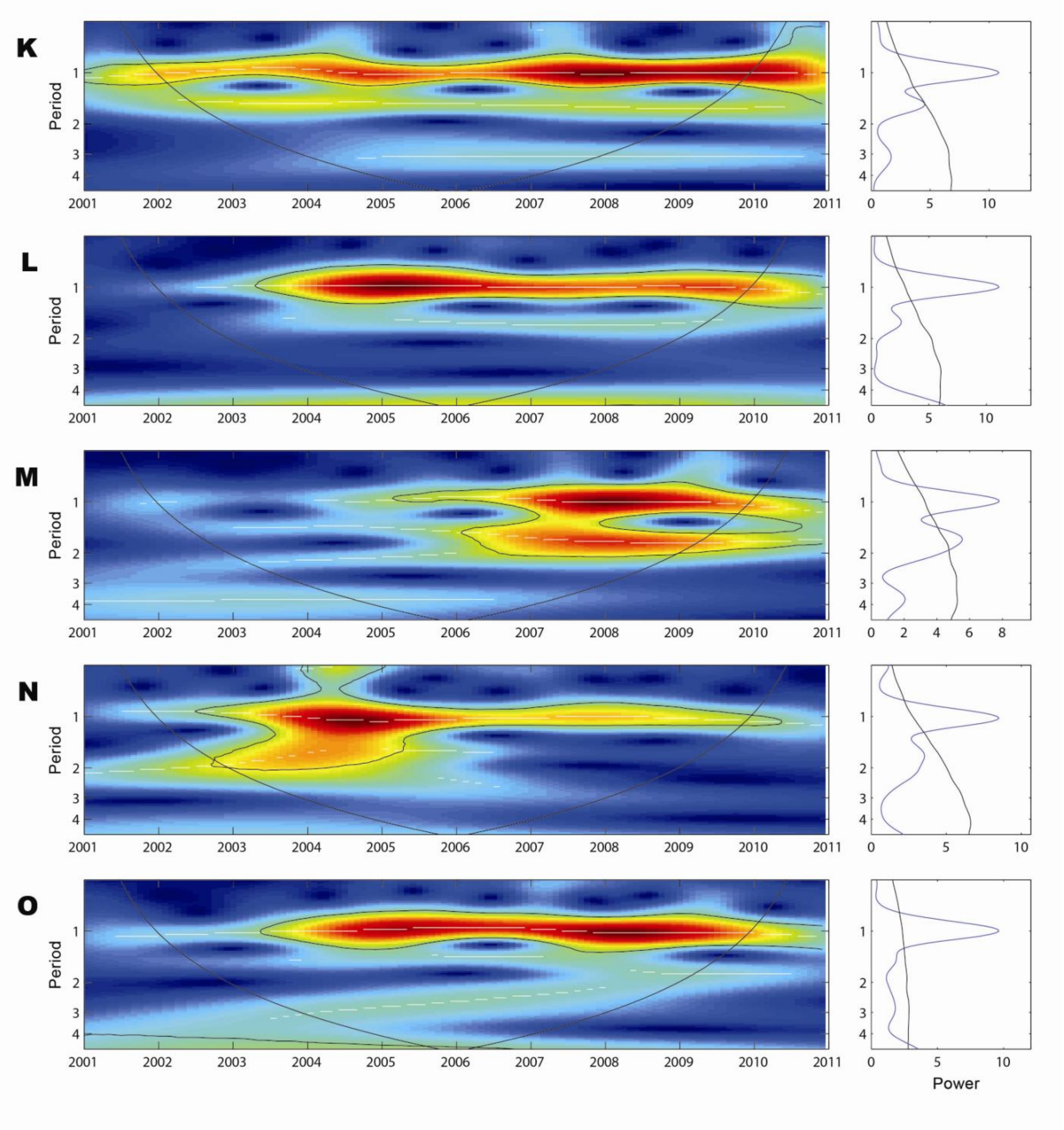
K
L
M
N
O
Period
1
2
3
4
2001
2002
2003
2004
2005
2006
2007
2008
2009
2010
2011
0
5
10
2
4
6
8
Power

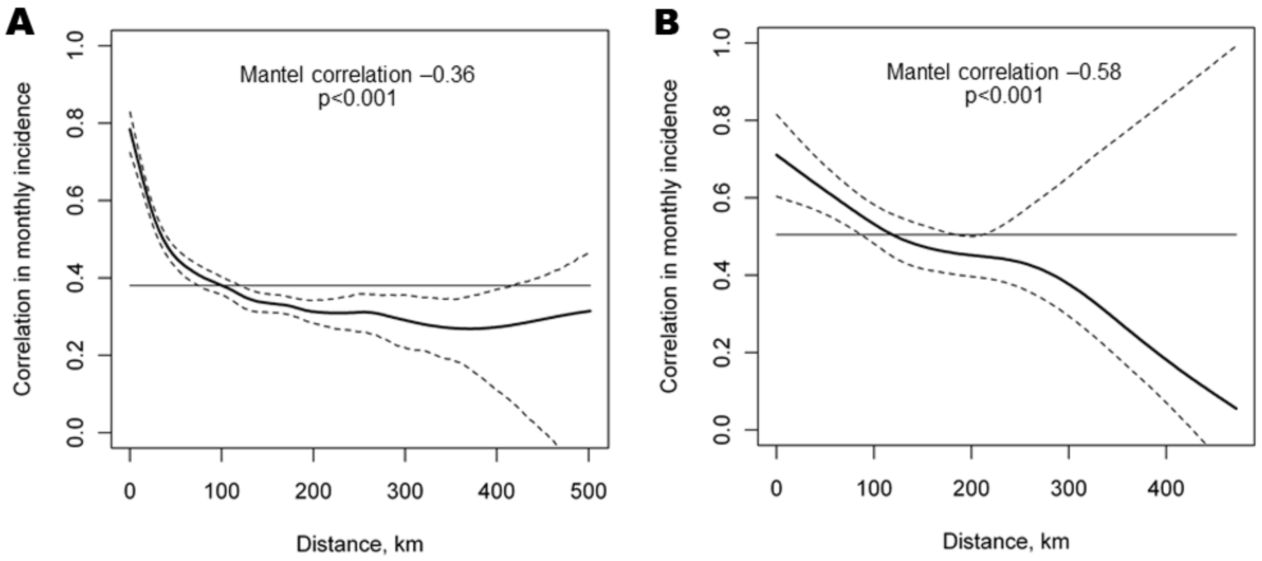

Technical Appendix Figure 3. Spatial synchrony in the magnitude and timing of annual dengue epidemics in southern Vietnam, at district (A) and province (B) level. Solid lines represent the synchrony between provinces/districts as a function of the distance between the centroids of those provinces/districts, in kilometers. Dotted lines represent the 95% CIs, and the horizontal line is the overall correlation across southern Vietnam. Synchrony was measured by pairwise correlation between provinces/districts in their standardized square root–transformed monthly incidence time series.
